# Vaccination with a BCG Strain Overexpressing Ag85B Protects Cattle against *Mycobacterium bovis* Challenge

**DOI:** 10.1371/journal.pone.0051396

**Published:** 2012-12-10

**Authors:** Caroline Rizzi, María Verónica Bianco, Federico Carlos Blanco, Marcelo Soria, María José Gravisaco, Valeria Montenegro, Lucas Vagnoni, Bryce Buddle, Sergio Garbaccio, Fernando Delgado, Karen Silva Leal, Angel Adrián Cataldi, Odir Antônio Dellagostin, Fabiana Bigi

**Affiliations:** 1 Núcleo de Biotecnologia, CDTec, Universidade Federal de Pelotas, Pelotas, Brazil; 2 Instituto de Biotecnología, CICVyA-INTA, N. Repetto y De los Reseros, Buenos Aires, Argentina; 3 Microbiología Agrícola, Facultad de Agronomía, Universidad de Buenos Aires, INBA-CONICET, Ciudad de Buenos Aires, Argentina; 4 Instituto de Patobiología, CICVyA- INTA, N. Repetto y De los Reseros, Buenos Aires, Argentina; 5 AgResearch, Hopkirk Research Institute, Palmerston North, New Zealand; University of Delhi, India

## Abstract

*Mycobacterium bovis* is the causative agent of tuberculosis in cattle but also infects other animals, including humans. Previous studies in cattle have demonstrated that the protection induced by BCG is not complete. In order to improve the protection efficacy of BCG, in this study we overexpressed Ag85B in a BCG Pasteur strain, by using an expression system based on the use of an auxotrophic strain for the leucine amino acid, and complementation with *leuD*. We found that vaccination of cattle with BCG overexpressing Ag85B induced higher production of IL-17 and IL-4 mRNA upon purified protein derivative (PPDB) stimulation of peripheral blood mononuclear cells (PBMCs) than vaccination with BCG. Moreover, the IL-17 mRNA expression after vaccination negatively correlated with disease severity resulting from a subsequent challenge with *M. bovis*, suggesting that this cytokine is a potential biomarker of cattle protection against bovine tuberculosis. Importantly, vaccination with the recombinant BCG vaccine protected cattle better than the wild-type BCG Pasteur.

## Introduction

Bovine tuberculosis (bTB) is not only a serious animal and zoonotic disease that causes significant financial loss but also a public health hazard. While the main host of *Mycobacterium bovis*, the causative agent of bTB, is cattle, other animals, including humans, may also be affected. Infection in humans occurs when unpasteurized milk (or derivatives) is consumed or when people are in contact with infected cattle. bTB is a factor that undermines the development of the dairy and meat industry and international commerce. Therefore, it is essential to control and eradicate this disease and an efficient vaccination strategy would help in the control of bTB. Vaccination of cattle to control bTB is particularly demanding in high-prevalence zones where it is economically unfeasible to slaughter animals [1].

Although previous studies with BCG in cattle have demonstrated reductions in disease severity after experimental challenge with virulent *M. bovis* strains, the protection induced by BCG was, in general, not complete [2]–[4]. The heterologous prime-boost strategy has shown to be the most promising approach to protect cattle against *M. bovis* challenge, and the combinations of BCG–DNA [5]–[7], BCG–protein [8], [9] and BCG–virus vectored vaccines [10] have been shown to induce better protection than BCG alone. However, some of these vaccination schemes demand an expensive production of booster vaccines and require additional handling of herds than single-dose vaccination schemes. The latter is particularly important in extensive breeding herds. In this study, we explored the attractive alternative of improving the protective efficacy of the BCG vaccine. To this purpose, we created a recombinant BCG strain that overexpresses Ag85B. The 85B antigen is a member of a protein complex commonly known as the Fbp complex (Ag85), which includes Ag85A, Ag85B and Ag85C [11]. These proteins are encoded by the *fbpA*, *fbpB* and *fbpC2* genes, which are located in different genomic regions. The Fbp complex is the main secreted protein constituent of mycobacterial cell culture and is also found in association with the bacterial surface [11], [12]. These proteins play an essential role in the pathogenesis of tuberculosis, and their main contribution to the virulence of *M. tuberculosis* is due to their physiological role in the synthesis of cell wall lipids [13], [14]. Both Ag85A and Ag85B have been shown to be among the most potent antigens identified [15], [16]. When expressed in a variety of delivery systems, these antigens have been shown to significantly improve the protection conferred by candidate vaccines against tuberculosis in animal models [17]. In this study, we overexpressed Ag85B in a BCG Pasteur strain, by using an expression system based on the use of a auxotrophic strain for the leucine amino acid, and complementation with *leuD* inserted into the plasmid vector (pUP410) [18]. This approach has two main advantages: it provides active selection *in vivo*, unlike antibiotic resistance markers, and it abolishes the need for using an antibiotic resistance gene as a vector component [18]. The protection efficacy of the resulting Δ*leuD* BCG-85B candidate vaccine against *M. bovis* challenge as well as the immune responses induced by the vaccine were tested in cattle and the results were compared with those obtained with BCG vaccination. We demonstrated that the recombinant BCG vaccine protects cattle better than the wild-type BCG Pasteur.

## Materials and Methods

### Ethics Statement

Animal experimentations were performed inside the biosafety facilities of the National Institute of Agricultural Technology (INTA), Argentina, in compliance with the regulations of Institutional Animal Care and Use Committee (CICUAE) of INTA and authorized by the National Service of Agricultural and Food Health and Quality (SENASA) and National Consultant Commission of Agricultural Biotechnology (CONABIA). Ethical approval for the study was obtained from CICUAE (n° 18/2011).

### Bacterial Strains and Culture Media

*Mycobacterium bovis* BCG strains were grown in Middlebrook 7H9 medium supplemented with 0.05% Tween 80 or in Middlebrook 7H10. Middlebrook media were supplemented with oleic acid-albumin-dextrose-catalase (OADC - Difco), 0.05% of Tween 80 and 0.2% of glycerol. When required, the L-leucine (Sigma-Aldrich, St. Louis, Missouri, USA) was added to a final concentration of 100 µg/ml. The *Escherichia coli* strain TOP10 (Invitrogen, Carlsbad, CA, USA) was used for cloning and was grown in Luria-Bertani medium at 37°C with addition of kanamycin 50 µg/ml.

### Cloning of the *fbpB* Gene and Construction of BCG Δ*leuD* Expressing Ag85B

The coding sequences for the Ag85B antigen were amplified from the *fbpB* gene of *M. bovis* genomic DNA. Primers used for *fbpB* gene PCR amplification were based on the complete *M. bovis* AF2122/97 genome sequence (5′-GGGGTACCCGCTATGTAGCTCCAATTC-3′ and 5′- GGGGTACCTCAGCCGGCGCC-3′), and were designed using Vector NTI 10.0 (Invitrogen, Carlsbad, CA, USA). Both forward and reverse primers contain restriction sites for *Kpn*I. The *M. bovis* 30-kDa antigen gene cassette, consisting of the *fbpB* gene coding region and its endogenous promoter (1500 bp), were obtained using standard PCR conditions and the enzyme Go Taq® Hot Start Polymerase Sample (Promega, Madison, Wiscosin, USA). The PCR product was digested with the *Kpn*I enzyme (Promega, Madison, Wiscosin, USA), and inserted into the pUP410 vector, which had been previously digested with the same restriction enzyme. Competent *E. coli* was then transformed with the recombinant plasmid (pUP410::*fbpB*) and the clones were checked by restriction enzyme digestion and PCR. To remove the kanamycin resistance gene by digestion, pUP410::*fbpB* was digested with *Hin*dIII and re-ligated with T4 DNA ligase (Invitrogen, Carlsbad, CA, USA).

*Mycobacterium bovis* BCG Pasteur Δ*leuD* electrocompetent cells were transformed with the product of ligation (pUP410::*fbpB* ΔkanR) and recombinant strains were selected in 7H10 media without L- leucine. Recombinant BCG were grown for 5 days in selective 7H9 media, the cells were harvested by centrifugation, and the total proteins were obtained by disruption using a ribolyser (Hybaid, Kalletal, Germany). The filtered culture supernatant was concentrated by precipitation with saturated ammonium sulfate solution. Proteins were fractionated on sodium dodecyl sulphate (SDS) −15% polyacrylamide gel and transferred to nitrocellulose membrane (GE Healthcare Live Sciences, Little Chalfont, Buckinghamshire, UK). Blots were probed with a mouse polyclonal antibody anti-Ag85B. Peroxidase-conjugated anti-mouse immunoglobulin G (Sigma-Aldrich, St. Louis, Missouri, USA) was used at a dilution of 1∶6000. Detection was carried out using TMB Liquid Substrate System (Sigma-Aldrich, St. Louis, Missouri, USA).

### Cattle Infections

All the animals used in this study were negative for the tuberculin skin test and showed absence of *in vitro* gamma interferon (IFN-γ) response to both avian tuberculin PPD (PPDA) and bovine tuberculin PPD (PPDB) at the beginning of the experiments. The results shown in this study are part of a trial that included another candidate vaccine whose results will be separately published.

Three groups of five-six Holstein-Fresian calves (three-four months old) were inoculated subcutaneously in the side of the neck with 10^6^ colony forming units (CFU) of either Δ*leuD* BCG-85B Pasteur, or BCG Pasteur suspended in buffer phosphate saline (PBS) or PBS. Eight weeks after vaccination, animals were infected with a wild boar strain *M. bovis* 04-303 by intratracheal instillation of 10^6^ CFU as described previously [19]. This inoculation procedure was carried out by first anaesthetizing the calves with xylazine HCl (Rompun, Bayer, Germany; 0.1 mg/kg) intravenously and then inserting an 80 cm endotracheal tube into trachea. A cannula was inserted through the endotracheal tube. The 1.5 ml inoculum containing the *M. bovis* was injected through the cannula and flushed out with sterile saline equal to 1.5 times the volume of the dead space of the cannula.

### Post-mortem Examination

Sixteen weeks after infection, the calves were euthanized and then thin slices of lungs and lymph nodes of the head and pulmonary region were analysed looking for granuloma formations. Animals that presented macroscopic lesions were positive for either bacterial isolation, *IS6110*-PCR in tissues or both. Macroscopic lesions were scored as following: Lung lesion scores: 0, no lesions; 1, 1 to 9 lesions; 2, 10 to 29 lesions; 3, 30 to 99 lesions; 4, 100 to 199 lesions; 5, ≥200 lesions. Total lymph node lesion score per animal for individual nodes: 0, no lesions; 1, 1 to 19 small lesions (diameter, 1 to 4 mm); 2, ≥20 small lesions; 3, medium-size lesions (diameter, 5 to 9 mm); 4, large lesions (diameter, ≥10 mm). Theses scores have been previously established by Wedlock et al. [20].

Tissues were fixed in 10% formal saline and processed for histological examination following staining with hematoxylin and eosin. Histophathological lesions were scored as following: score 0: without pathological changes; score 1: pneumonia and peribronchial infiltration; score 2: most of granuloma type I; score 3: granuloma type I and II; score 4: most of granuloma type II, score 5: granulome type II and III; score 6: most of granuloma type III; score 7: granuloma type III and IV; score 8: most of granuloma type IV; score 9: granuloma type IV and cellular infiltration in bronchioles.

### Gamma Interferon (IFN-γ) Release Assay

Blood samples were taken both at the beginning of the experiment for evaluation of pre-immune status and at the following time points: 30 and 60 days post-vaccination, and 40, 70 and 100 days post-challenge. Heparinized blood samples were dispensed in 200 µl aliquots into individual wells of a 96-well plate. Wells contained whole blood plus 20 µg/ml *M. bovis* PPDB (Prionics, Shlieren, Zurich, Switzerland), 20 µg/ml *Mycobacterium avium* PPDA (Prionics, Shlieren, Zurich, Switzerland). Blood cultures were incubated for 18 h, and plasma was harvested and stored at −80°C. IFN-γ concentrations in stimulated plasma were determined using a commercial ELISA-based kit (Bovigam™; Prionics, Shlieren, Zurich, Switzerland). Absorbance of standards and test samples were read at 450 nm. The optical density (OD) for the PBS controls, which was usually approximately 0.1 OD units, was used to normalize individual readouts and to calculate the differential optical density indexes (ODIs), where the results were obtained by subtracting the results of the PBS-stimulated cultures from that for the antigen stimulated cultures were divided by the results of the PBS-stimulated cultures.

### Tuberculin Testing

All animals were tested for skin tuberculin test (DTH) both before and one month after vaccination, and three months after the *M. bovis* challenge. Animals were intradermally injected with 0.1 mg of PPDB and the thickness of the caudal fold tuberculin skin test was measured using callipers before and 72 h after injection. PPDB was obtained from the National Service of Agricultural and Food Health and Quality (SENASA, Buenos Aires, Argentina).

### Flow Cytometry and Cytokine mRNA Analysis

Blood samples were taken both at the beginning of the experiment for evaluation of the pre-immune status and at the following time points: 15, 30 and 60 days post-vaccination, and 20, 40, 70 and 100 days post-challenge. For cytokine analysis blood samples were taken only 15 and 30 days post-vaccination.

Heparinized blood (10 ml) from each animal was used for PBMC isolation by gradient centrifugation over Histopaque 1077 (Sigma-Aldrich, St. Louis, Missouri, USA) following the manufacturer’s protocol. PBMCs were incubated at 37°C in RPMI complete medium supplemented with 10% of bovine fetal serum (Internegocios, Mercedes, Buenos Aires, Argentina) and 20 µg/ml final concentration of PPDB (Prionics, Shlieren, Zurich, Switzerland) on 12-well tissue culture plates for 16 h for RNA extraction and 48 h for flow cytometry determinations.

For flow cytometry determinations, 2×10^6^ cells were incubated either with or without PPDB. To evaluate the expression of CD4 (MCA 1653A647, IgG2a), CD8 (MCA837PE, IgG2a) and CD25 (MCA2430F and MCA2430PE) surface markers, cells were stained with fluorescent-conjugated monoclonal antibodies (AdDSerotec, Oxford, UK).

Stained cells were analysed in a FACScalibur cytometer (BD, Franklin Lakes, NJ, USA) using Cell Quest software. Analysis gates were set on lymphocytes according to forward and side scatter. Expression of CD25 (IL-2R) was analysed in CD4+ and CD8+ populations. Percentages of IL-2R-expressing cells were calculated as the ratio of CD4+ or CD8+ cells expressing CD25 and total CD4+ or CD8+ cells. Mann-Whitney test was used for statistical analysis.

Total RNA was extracted from PPDB-stimulated PBMCs using a commercial kit (QIAGEN). The quality and quantity of RNA and the synthesis of cDNA were assayed as described previously [21]. The mRNA of cytokines (IL-2, IL-4, IL-10, IL-17, and IFN-γ) was quantified by qPCR by using specific primers [21]. qPCR results were analysed using the REST software as described previously [22]. For each animal, the pre-immune condition was used as the calibrator, and *gadph* were used as reference genes. Data were analysed using Mann-Whitney test. Correlation analysis between total gross pathology scores observed at necropsy and cytokine expression of individual animals were made at 15 and 30 days post-vaccination and at 20, 40, 60 and 100 days post challenge.

### Statistical Analyses

Statistical analysis was performed using GraphPad prism 5.03 software (GraphPad Software, San Diego California USA). Differences in pathology scores, DTH, cell activation and cytokine transcription were analysed using Mann-Whitney non parametric two-tailed *t* test. Differences in IFN-γ production was analysed using ANOVA test. Correlations between cytokine expression or Bovigam assay and the degree of pathology were assessed by linear regression analysis.

## Results

### Construction and Ag85B Overexpression of Recombinant BCG

**Figure 1:**
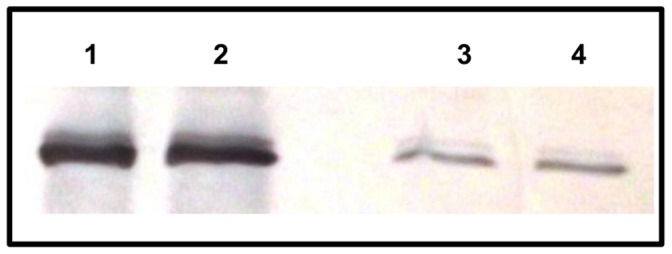
Western blot demonstrating Ag85B expression in BCG Pasteur Δ*leuD*. Lane 1, whole-cell lysates of rBCG transformed with pUP410::*fbpB*; lane 2, culture supernatant of rBCG transformed with pUP410::*fbpB*; lane 3, whole-cell lysates of wild-type BCG; lane 4, culture supernatant wild-type BCG.

The integrity of the recombinant vector pUP410::*fbpB* used in this study to over express Ag85B in BCG Pasteur was confirmed by PCR and restriction site analysis (data not shown). Recombinant protein was readily detectable in Δ*leuD* BCG-85B lysates and culture supernatants by Western blot using polyclonal anti-Ag85 antibody (Figure 1). Both the cytoplasm and the secreted recombinant protein presented 30 kDa of molecular mass, visually in greater quantities than in wild-type BCG Pasteur, which has relatively low levels of endogenous expression of Ag85B.

### Protective Efficacy of Δ*leuD* BCG-85B

Groups of calves were vaccinated with either BCG Pasteur or BCG Pasteur overexpressing Ag85B. A third group was left unvaccinated. Eight weeks after vaccination, calves were challenged intratracheally with *M. bovis*.

**Figure 2:**
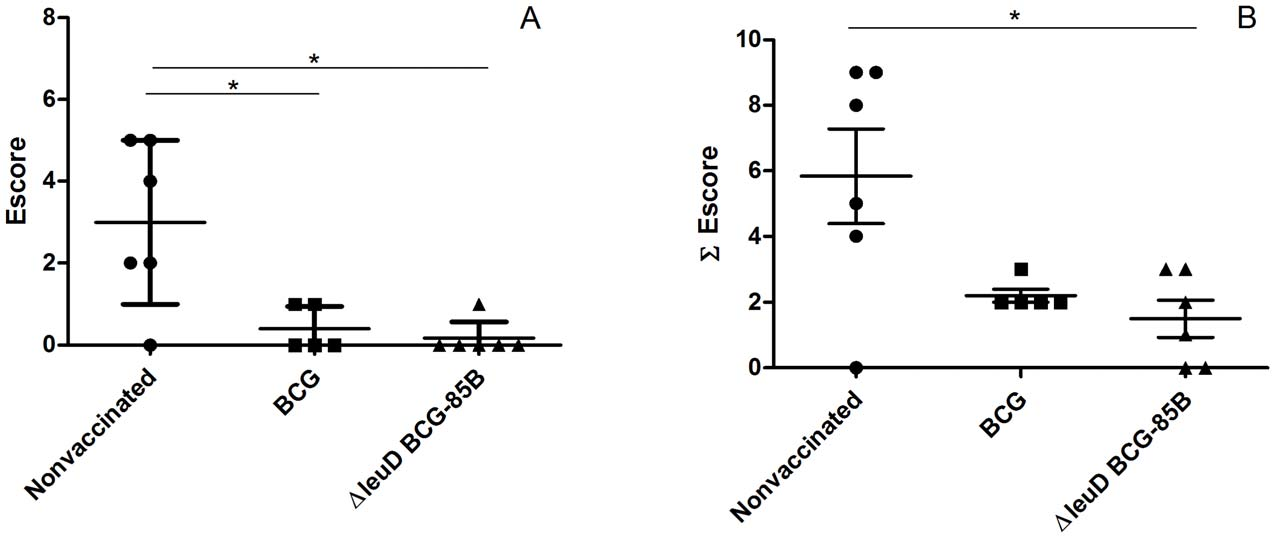
Protective efficacy as measured by gross pathology. The calves were euthanized sixteen weeks after infection and thin slices of lungs and lymph nodes were analysed looking for granuloma formations. Pathology scores were established using the scoring system described in Material and Methods (A) Mean pathology scores of lungs in vaccinated and nonvaccinated groups. (B) Mean pathology scores of total lesions (head lymph nodes, respiratory tract-associated lymph nodes and lungs) in vaccinated and nonvaccinated groups. Pathology scores for individual animals are plotted. Horizontal lines indicate median values. Significance was determined by Mann Whitney test: * Statistically significantly different, *P*<0.05.

The level of protection afforded by each strain of BCG was determined at 16 weeks post-challenge. Although all groups of animals presented typical tuberculosis lesions in lungs or lymph nodes, the number of lesions varied between groups. Although both vaccinated groups (Δ*leuD* BCG-85B and BCG) showed reduced pathology in the pulmonary lymph nodes compared to that of nonvaccinated animals, as judged by the number of lesioned lymph nodes, this parameter did not reach statistical significance (data not shown). In contrast, the median score for lesions in lungs in the groups vaccinated with either Δ*leuD* BCG-85B or BCG was statistically lower than that of the nonvaccinated group (Figure 2A, *P*<0.05). Importantly, the total pathology scores, which take into account disease severity in lungs and lymph nodes, were significantly reduced after Δ*leuD* BCG-85B vaccination but not after BCG vaccination, when compared to those for nonvaccinated animals (Figure 2B, *P*<0.05).

A histopathological analysis of lung lesions revealed that both vaccinated groups (Δ*leuD* BCG-85B and BCG) showed significantly reduced pathology when compared to that in lungs of nonvaccinated animals, as judged by the type of lesions. Moreover, the median score for lesions in lungs in the groups vaccinated with Δ*leuD* BCG-85B was statistically lower than that of the BCG-vaccinated group (*P*<0.05).

**Figure 3:**
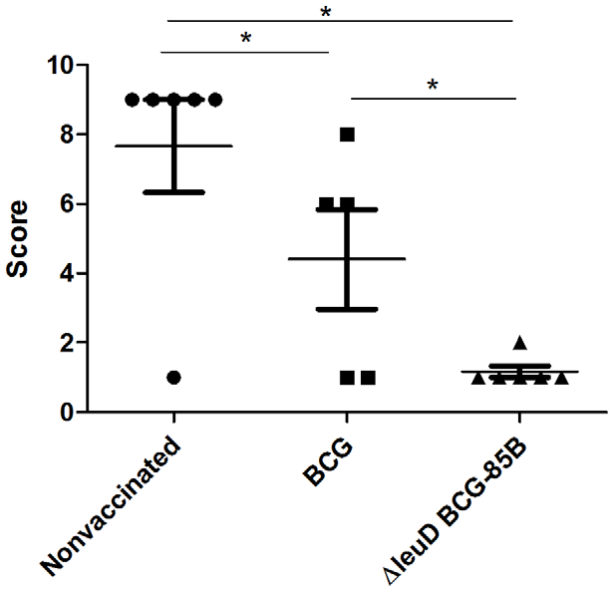
Protective efficacy as measured by histopathology in lungs. Histopathology scores in lungs of individual animals in vaccinated and nonvaccinated groups are plotted. Tissue samples from lungs and lymph nodes were obtained sixteen weeks after infection. Histopathology scores were established using the scoring system described in Material and Methods. Horizontal lines indicate mean values. Significance was determined by Mann Whitney test, * Statistically significantly different, *P*<0.05.

In detail, the nonvaccinated group presented numerous small, medium and large lesions in thoracic LN (Material S1). A single animal had a large number of small and medium lesions in head LN (data not shown). Five animals developed a variable number of macroscopic lung lesions: from 14 small lesions to large areas of confluent lesions, affecting whole lung lobes (Figure 2 and Material S2). This group presented lung lesions with a high prevalence of type IV granulomas and these were surrounded by type I granulomas indicating the active proliferation of *M. bovis* and abundant cellular infiltration in the bronchioles (Figure 3 and Material S2). In the BCG vaccinated group only two animals exhibited lesions in lungs (2 and 17 lesions) with sizes ranging from 2-10 mm in diameter (Figure 2 and Material S2). The lesions contained type III granulomas, some type IV granulomas surrounded by abundant type I granulomas and pneumonia (Figure 3 and Material S2). All animals showed small lesions in lung LN (6-100 lesions/LN) (Material S1). A single animal had 7 small lesions in head LN (data not shown). Finally, in the Δ*leuD* BCG-85B-vaccinated group a single animal had one macroscopic lung lesion of 5 mm with small type I granulomas (Figure 2, 3 and Material S2). Four animals showed small lesions in thoracic LN with granulomas type I, III and IV, and no lesions in head LN were observed. Taken together, these results indicate that Δ*leuD* BCG-85B protects cattle better than BCG, as judged by the severity of the lesions in lungs and lymph nodes.

### Blood IFN-γ Responses after Vaccination and Challenge

**Figure 4:**
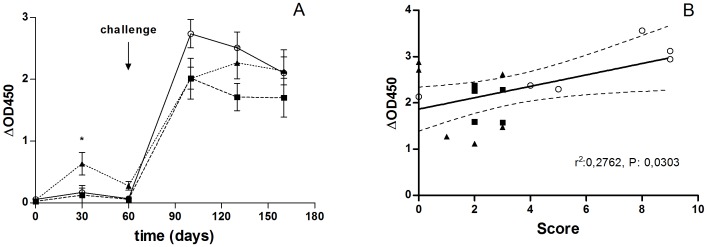
IFN-γ production in PPDB-stimulated PBMCs. (A) IFN-γ responses in vaccinated and nonvaccinated animals following vaccination and after challenge. IFN-γ production was measured in PPDB-stimulated blood from animals vaccinated with BCG (squares), Δ*leuD* BCG-85B (triangles) or nonvaccinated (circles) at different time points (0, 30 and 60, 120 and 150 days post-vaccination). Animals were infected eight weeks after vaccination and sacrificed sixteen weeks post-challenge. Arrow indicates the challenge time point. Significance was determined by ANOVA test. Statistically significantly different to that for the nonvaccinated group, *P<0.05. (B) Correlation between IFN-γ production in PPDB-stimulated PBMCs and disease severity. Results are expressed as IFN-γ 40 days post-challenge of individual animals in relation to the corresponding total gross pathology scores. Solid line indicates linear regression; dashed lines indicate 95% confidence intervals. Values for *r^2^* and *p* of linear regression analysis are indicated.

IFN-γ production after *in vitro* stimulation with purified protein derivative (PPDB) was monitored in blood samples from all animal groups at different points after vaccination and challenge. At 30 post-vaccination, only blood samples from the Δ*leuD* BCG-85B -vaccinated group responded with significantly higher IFN-γ production (*P*<0.05) than that from blood samples from the nonvaccinated group (Figure 4A). Post-challenge, all animal groups displayed strong IFN-γ responses, and no significant differences were detected among them from this point on (Figure 4A).

We compared the IFN-γ production in whole-blood cultures after vaccination and challenge in relation to the pathology found at post-mortem. We found a statistically significant positive correlation (*P* = 0.023) between IFN-γ production upon PPDB stimulation at 40 days post-challenge and disease severity as judged by macroscopic analysis of lesion (scores) in lungs and lymph nodes following challenge (Figure 4B). There was no statistically significant correlation between IFN-γ production and disease severity in other time points assessed.

### Skin Test Reactivity in Vaccinated and Nonvaccinated Calves after Vaccination and Challenge

**Figure 5:**
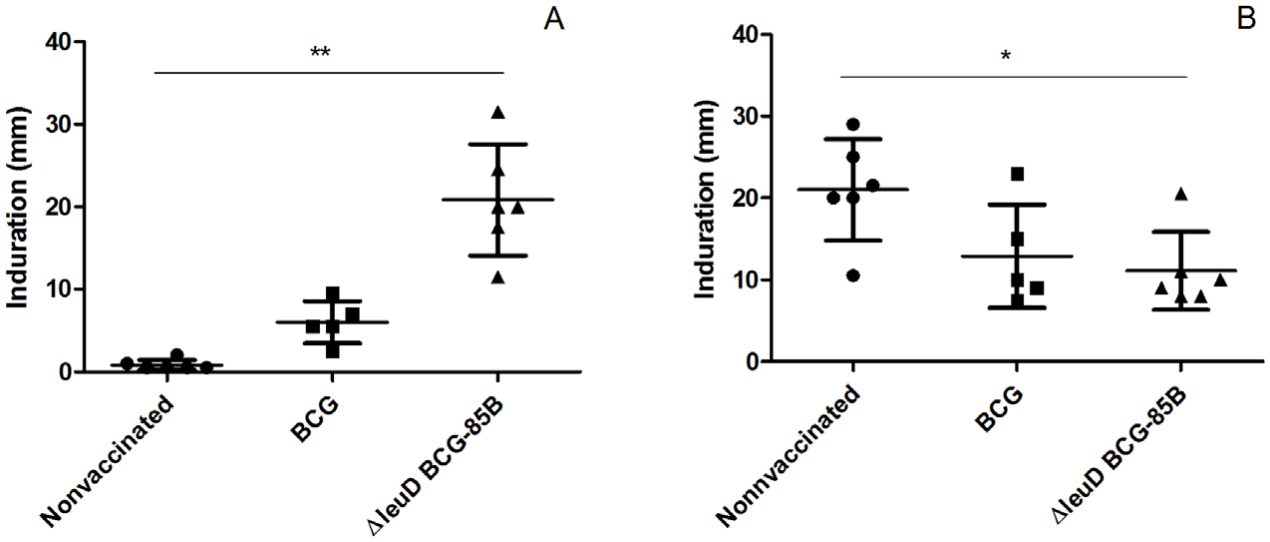
Mean tuberculin skin test responses to PPDB. Animals were vaccinated with BCG (n = 5, squares), Δ*leuD* BCG-85B (n = 5, triangles) or inoculated with PBS (n = 6, circles) and challenged after eight weeks with *M. bovis*. Values indicate skin thickness at one month post-vaccination and prior to challenge (A), and three months post-challenge (B). Horizontal lines indicate mean values. Data were analysed using a Mann Whitney test,*P<0.05, **P<0.005.

Skin test reactivity was evaluated after vaccination and challenge in all cattle groups. Calves vaccinated with either BCG or Δ*leuD* BCG-85B reacted to PPDB antigen at one month post-vaccination, while all animals in the nonvaccinated group were negative for this test. The mean skin thickness for the BCG group was statistically lower than that of the Δ*leuD* BCG-85B group (*P*<0.005), prior to *M. bovis* challenge (Figure 5A). However, upon sacrifice (three months post challenge), only the response to PPD in the Δ*leuD* BCG-85B group was significantly lower than in the nonvaccinated group (Figure 5B, *P*<0.05). No correlation was detected between skin test reactivity and disease severity, as judged by scores of total lesions (data not shown).

### Activation of CD4+, CD8+ in PPDB after Vaccination and Challenge

**Figure 6:**
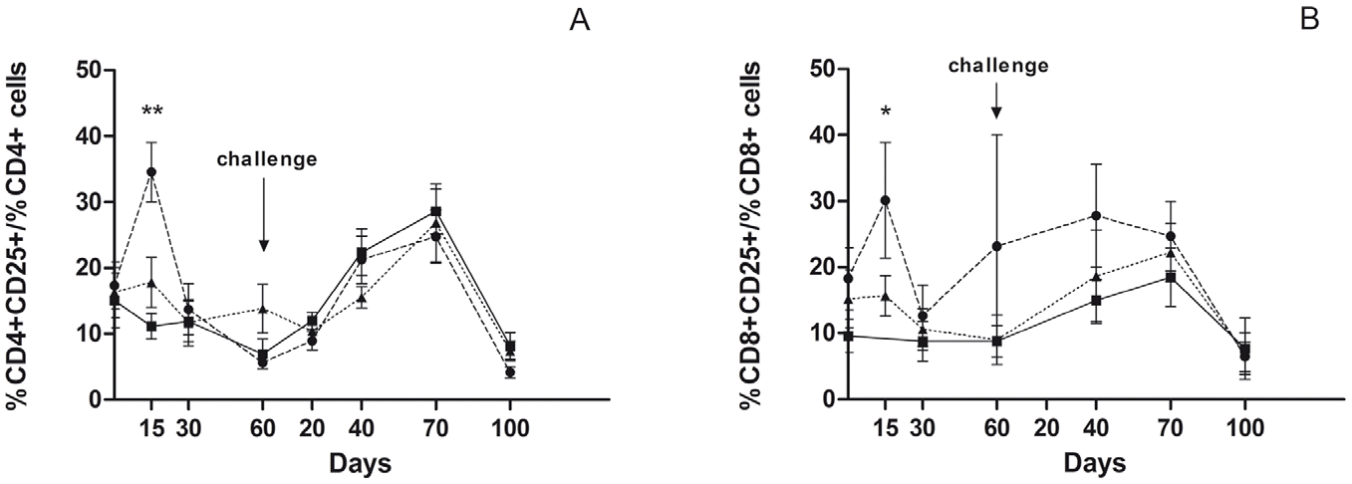
Determination of lymphocyte subsets in PPDB-stimulated PBMCs. Percentages of lymphocyte cell subsets CD4+ (A) and CD8+ (B) expressing CD25 for PPDB stimulated-PBMCs from animals vaccinated with BCG (n = 5, triangles), Δ*leuD* BCG-85B (n = 6, circles) or nonvaccinated (n = 6, squares) at 15, 30 and 60 days after vaccination and 20, 40, 70 and 100 days after challenge. The arrow indicates the challenge time point. Data were analysed using Mann-Whitney test for comparison between groups. (Statistically significantly different to that for the nonvaccinated group *P<0.05 and ** *P*<0.01).

The recall response to PPDB of lymphocyte subsets in vaccinated and nonvaccinated animals was evaluated after vaccination and challenge. At two week post-vaccination, the percentages of CD4+ T and CD8+ T cells expressing CD25 in Δ*leuD* BCG-85B -vaccinated calves peaked significantly (*P*<0.01 and *P*<0.05, respectively) upon stimulation with PPDB as compared to those in nonvaccinated calves (Figure 6A and B). In contrast, the mean percentages of CD4+ T and CD8+ T cells in the BCG-vaccinated group were equivalent to those of nonvaccinated animals. After challenge, the percentages of both CD4+ and CD8+ T cells upon stimulation were similar in animal groups, peaking at 70 days post-challenge. Importantly, in peripheral blood mononuclear cells (PBMCs) from animals before vaccination, the percentages of CD4+ and CD8+ cells were not significantly altered upon PPDB stimulation, indicating that the animals used in this study were neither previously sensitized nor infected with *M. bovis* (Figure 6).

### Expression of Cytokines in PBMCs after Vaccination and Challenge

**Figure 7:**
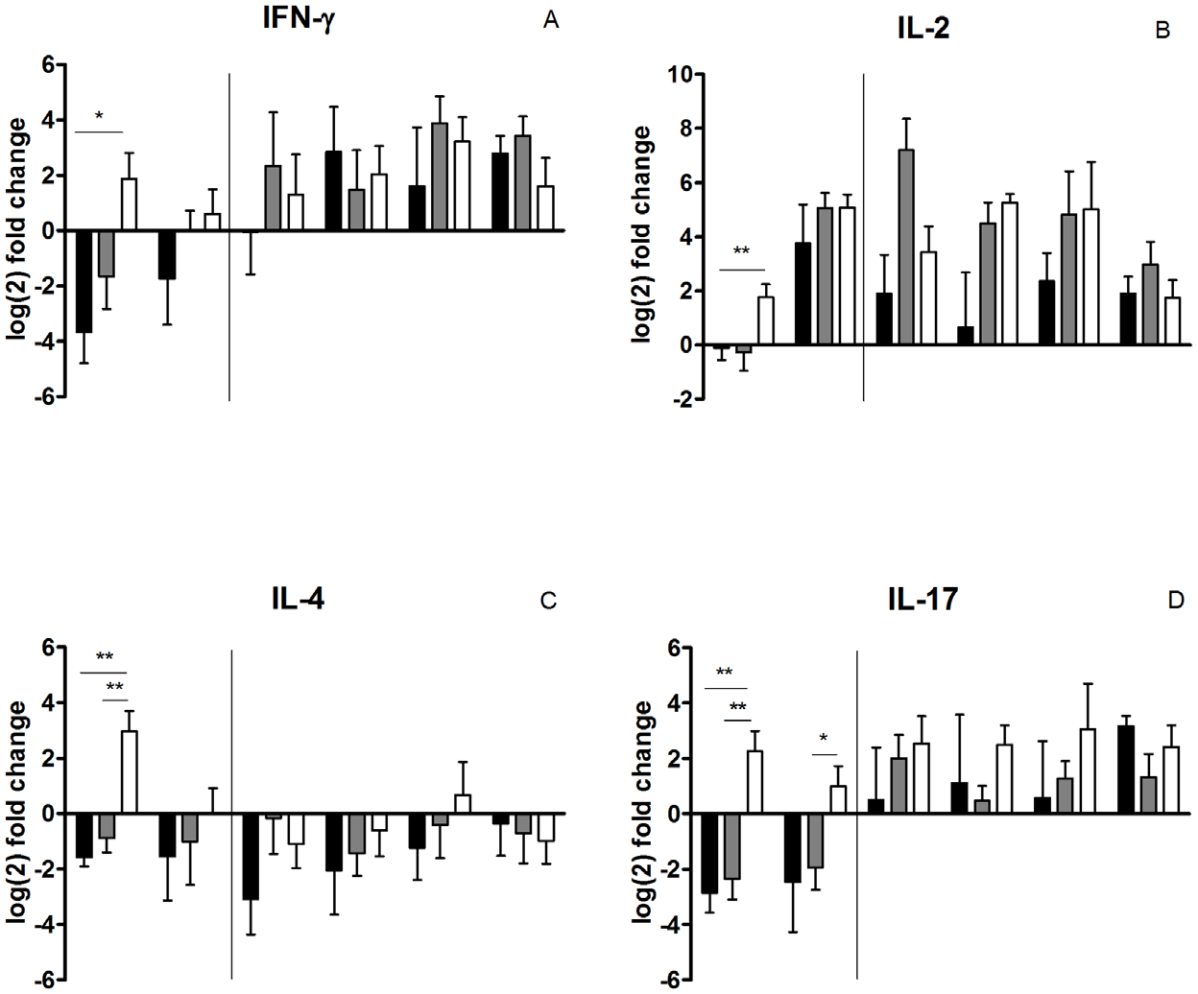
Relative cytokine gene expression. Gene expression of IFN-γ (A), IL-2 (B), IL-4 (C) and IL-17 (D) was measured in PPDB-stimulated PBMCs from animals vaccinated with BCG (gray), Δ*leuD* BCG-85B (white) or nonvaccinated (black) at different time points (15 and 30 days post-vaccination and 20, 40, 70 and 100 days post-challenge). Transversal line indicates the challenge time point. Relative gene expression was calculated using the 2-ΔΔCt method with E correction, using *gadph* mRNA expression as reference gene and the pre-immune condition as the calibrator. Data were analysed using Mann-Whitney test, statistically significantly different, * *P*<0.05 and ** *P*<0.01. The bars indicate the median fold change.

In order to compare the immune response profile of cattle vaccinated with the candidate vaccine Δ*leuD* BCG-85B with that of animals vaccinated with BCG and that of the nonvaccinated group, we measured the cytokine mRNA levels after stimulating the cells with PPDB at different points after vaccination and challenge (Figure 7). Values for sequential samples were normalized to values before inoculation for each animal. We assessed the expression of the proinflammatory cytokines IFN-γ and IL-2 and the anti-inflammatory cytokines IL-4 and IL-10. Given that IL-17 expression has been proposed as a biomarker of cattle protection against bovine tuberculosis [9], we also quantified the mRNA level of this cytokine.

At two weeks post-vaccination, only the group vaccinated with Δ*leuD* BCG-85B responded to PPDB stimulation with significant production of both IFN-γ and IL-2 mRNA as compared to the values for the nonvaccinated group (*P*<0.05 and *P*<0.001, respectively) (Figure 7A and 7B). This high expression of IL-2 mRNA is consistent with the highest frequency of CD4+ and CD8+ cells expressing the IL-2 receptor (IL-2R) detected at two weeks post-vaccination in stimulated PBMCs of the Δ*leuD* BCG-85B -vaccinated group (Figure 6). After one month of vaccination, the expression of IL-2 in PBMCs from both vaccinated groups increased upon stimulation compared to pre-vaccination, but, surprisingly, the PBMCs of the nonvaccinated calves also responded to the PPDB stimulation with IL-2 mRNA expression at this time point (Figure 7B).

Stimulation with PPDB induced greater IL-4 gene expression in animals vaccinated with Δ*leuD* BCG-85B than in those vaccinated with BCG at two weeks post-vaccination (*P*<0.01) (Figure 7C), while the expression of IL-10 did not change upon stimulation of PBMCs from all groups, along the experiment (data not shown).

Importantly, at two and four weeks post-vaccination, PBMCs obtained from cattle vaccinated with Δ*leuD* BCG-85B responded to PPDB stimulation by expressing 24.2 fold and 7.6 fold, respectively, more IL-17 mRNA than cells from animals vaccinated with BCG (*P*<0.01) (Figure 7D). In fact, stimulation of cells from the BCG group did not induce IL-17 production at any point time after vaccination or prior to challenge.

**Figure 8:**
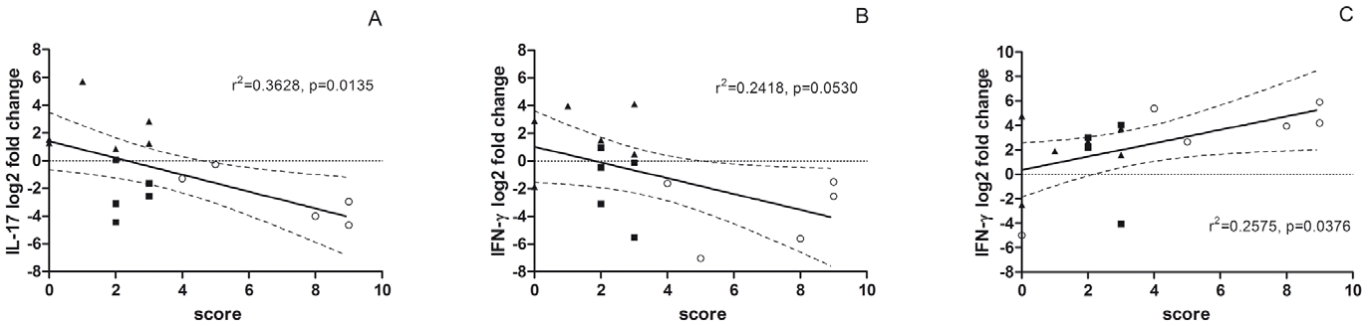
Correlation between IL-17 and IFN-γ mRNA expression in PPDB-stimulated PBMCs and disease severity. Results are expressed as mean increases in IL-17 (A) and IFN-γ (B) transcription at 15 days post-vaccination and IFN-γ transcription at 40 days post-challenge (C) of individual animals in relation to the corresponding total gross pathology scores (head lymph nodes, respiratory tract-associated lymph nodes and lungs). Animals vaccinated with either BCG (squares) or Δ*leuD* BCG-85B (triangles) and nonvaccinated (circles) were infected eight weeks after vaccination and sacrificed sixteen weeks post-challenge. Solid lines in panels A and B indicate linear regression; dashed lines indicate 95% confidence intervals. Values for *r^2^* and *p* of linear regression analysis are indicated.

Extending our analysis, we compared IL-17 and IFN-γ expression in relation to the pathology found at post-mortem. We found a statistically significant negative correlation (*P* = 0.0178) between IL-17 mRNA expression upon PPDB stimulation at two weeks post-vaccination and disease severity (pathology scores) (Figure 8A).

The expression of IFN-γ mRNA before challenge showed a weak not statistically significant correlation with disease severity (Figure 8B). However, at 40 days post-challenge, the expression of IFN-γ mRNA positively correlated with the presence of lesion in organs (*P* = 0.0231) (Figure 8C). After challenge, the expression of all other cytokines upon PPDB stimulation was equivalent in all groups.

## Discussion

Ag85B has shown to induce protection in both murine and guinea pig models of infection when expressed in a wide variety of vectors such as: rBCG, vaccines based on recombinant modified Vaccinia virus Ankara (MVA) and DNA-based vaccines. Indeed, MVA-Ag85A is one of the tuberculosis vaccines that are currently undergoing human clinical trials [15]. A BCG Tice strain overexpressing Ag85B has been shown to protect guinea pigs against a *M. tuberculosis* challenge at higher level than its parental strain [23], [24]. Furthermore, in mice, overexpression of Ag85B, Ag85A and TB10.4 antigens in a recombinant BCG expressing perfringolysin O has shown to induce better protection than the parental BCG [25]. In agreement with these previous findings and with the immunogenic properties of the Ag85B antigen, in this study we found that overexpression of the Ag85B antigen in Δ*leuD* BCG Pasteur clearly improved its vaccine efficacy. Indeed, significant reduction in the histological damage of lungs was induced in Δ*leuD* BCG-85B-vaccinated calves as compared to that induced by vaccination with BCG. However, in order to confirm the improved efficacy of this vaccine candidate the study should be extended to a larger number of calves.

The novelty of this work is the use of auxotrophic complementation system as selectable marker for stable expression of 85B antigen in BCG. Although other studies have demonstrated the use BCG overexpressing 85B, the replicating vectors employed have antibiotics used as selection markers [24], [26], [27]. Epissomal vectors provide higher expression level of heterologous proteins than integrative vectors, but the stability of multicopy vectors in BCG depends on selective pressure normally associated with resistance to antibiotics [28]. This type of selective pressure is lost during *in vivo* BCG replication, and results in loss of the epissomal vector. This might be due to a counter-selective pressure within the macrophage on strain expressing genes that are not part of the endogenous mycobacterial chromosome [29]. Méderlé et al demonstrated decreased immune response in recombinant BCG that rapidly lost the plasmid *in vivo* compared to stable strains carrying the heterologous gene integrated into the bacterial genome [30]. Stable expression of the recombinant Ag85B gene was obtained in Δ*leuD* BCG-85B due to selective pressure provided by the auxotrophic complementation system [18]. This is based on the fact that only the complemented Δ*leuD* BCG strain is able to grow inside macrophages [31]. Our system showed 100% stability *in vivo* for over 30 weeks in mice [18] and for over 20 weeks in hamsters [28], a feature not demonstrated by Badell et al. [27] when only 54% of *in vivo* BCG recovered after 58 days were resistant to antibiotics. An additional advantage is the absence of an antibiotic resistance gene, a requirement for a live bacterial vaccine that has been ignored in most experimental studies with recombinant BCG [20].

Given that there is no doubt about the essential protective role of CD4+ and CD8+ T cells to control tuberculosis infections, it is important that any candidate vaccine be capable of stimulating these cell populations. Therefore, the most effective vaccination strategies will be those that stimulate CD4+ and CD8+ T cell responses to produce Th1-associated cytokines. In accordance with these requirements, the candidate Δ*leuD* BCG-85B vaccine elicited stronger T-cell responses in cattle, with activation of CD4+ and CD8+ following stimulation with the *M. bovis* specific antigen PPDB, than that of the nonvaccinated group, while this was not seen for the BCG-vaccinated group.

Vaccination of cattle with Δ*leuD* BCG-85B induced in stimulated PBMCs, prior to *M. bovis* challenge, the transcription of IFN-γ, IL-4 and IL-17 mRNAs, while vaccination with BCG did not. This higher IFN-γ production in Δ*leuD* BCG-85B vaccinated cattle as compared to BCG-vaccinated and nonvaccinated groups was also detected in whole-blood PPD-B stimulated samples. However, the overall level of IFN-γ detected in individual animals before challenge did not correlate with protective efficacy, which is consistent with previous TB vaccine studies in cattle (reviewed by Buddle et al. [32]). Altogether these results indicate that the use of IFN-γ production as predictor of cattle protection against *M. bovis* challenge deserves more investigation. The higher IL-4 mRNA expression induced in the Δ*leuD* BCG-85B -vaccinated group may suggest that anti-inflammatory responses in animals inoculated with this candidate vaccine were generated to attenuate the adverse effect of an exacerbated inflammation of the host tissues driven by the highly expressed IFN-γ.

Importantly, we found a negative correlation between IL-17 expression after vaccination and the disease severity observed post-mortem. These results confirm the findings of Vordermeier et al. [10], who reported that high IL-17 responses in vaccinated cattle prior to challenge correlates with protection against *M. bovis*, and validate the use of this cytokine expression as a predictor or surrogate of protection, which would be very useful to estimate the vaccine efficacy without the need to infect animals with *M. bovis*.

It has been reported that in cattle experimentally infected with *M. bovis* the expression of IFN-γ, TNF-α, iNOS and IL-4 by PBMCs increase in response to infection, andthat animals with high pathology express more IFN-γ, TNF-α, iNOS and IL-4 than animals with low pathology [33]. However, the source of IFN-γ production seems to have relevance in its association with pathology since previous studies in cattle have only found significant correlations between pathology and post-challenge IFN-γ secretion when PBMC cultures were stimulated with ESAT-6/CFP10 but not when these cultures were stimulated with PPDB [3], [34]. In the current study, we found positive correlation between the expression of IFN-γ in PPDB-stimulated PBMCs and the extent of tissue damage, at 40 days post-challenge, at both transcriptional and translational levels, but, after 40 days post-challenge, this positive correlation was no longer detected. These results suggest that PPD-B specific IFN-γ secretion from PBMC cultures is a marker of diseases severity, although the inclusion of *M. bovis* specific antigen, such as ESAT-6/CFP-10, would improve the power of this disease predictor.

No correlation was observed between IL-4 mRNA expression and disease severity (data not shown). In fact, the expression of IL-4 mRNA was practically unchanged after *M. bovis* challenge in all cattle groups. Similarly, no significant changes were detected in the expression level of IL-10 mRNA upon *M. bovis* infection or vaccination for any cattle group. It has been proposed that as the tuberculosis disease progresses, there is a shift from Th1 to Th2 responses. Although our results suggest that the immune profile did not shift during the experimental time of this study, we cannot rule out the possibility that it may occur later during the infection.

Taken together, these data indicate that, in cattle, Δ*leuD* BCG-85B induces a strong Th1 response that is maintained for at least 60 days. Nevertheless, it will be of interest in further studies to determine which kind of T cell subpopulation produces each cytokine and identify those T cell populations that simultaneously produce more than one cytokine, as recent studies in mice have associated vaccine success with the increased presence of so-called polyfunctional T cells that produce, for example, IL-2, IFN-γ, and TNF-α [35]–[37]. The lack of commercial reagents to detect IL-2 and some other cytokines has been an important limitation toward the study of polyfunctional CD4 T cells in cattle as to detect intracellular cytokines by flow cytometry in cattle. However, an antibody that recognizes biologically active bovine IL-2 has been recently identified [38]; therefore the availability of this new tool could allow a better understanding of the complex T cell-mediated immune responses in cattle. It would be also important to establish the frequencies of memory T cell subsets that proliferate in response to specific stimulation in Δ*leuD* BCG-85B vaccinated animals. Memory T cells can be divided in central memory T cells (T_CM_) and effector memory T cells (T_EM_) according to their homing characteristics and effector function, and increasing evidence indicates that T_CM_ have a greater capacity than T_EM_ in mediating protection because of their high proliferative responses [39]. This analysis would allow us to dissect the T cell responses induced post-challenge that may explain the lack of correlation observed when the level of protection conferred by vaccination is compared to T cell frequencies, in total, without discriminating T cell subsets.

Altogether these results demonstrate that vaccination of cattle with Δ*leuD* BCG-85B induces protective immune responses against TB. However, some immunological parameters, such as cytokine expression and T cell proliferation, indicate that this protective immune response is only significantly superior to that induced by conventional BCG, early after vaccination. Therefore, we speculate that use of a prime boost scheme, in which viral vectors expressing antigen 85B boosts the anti-85B response induced by Δ*leuD* BCG-85B vaccine, will significantly improve the efficacy of the candidate here described. The study used out-bred animals as this is relevant to the use of TB vaccines in the field. Although there have been some studies investigating natural resistance to TB in cattle there have not been reports on the influence of genetic background of cattle to their response to vaccination against TB.

It has been clearly demonstrated that the specificity of the tuberculin skin test, used for the diagnosis of bTB, is compromised by vaccination with vaccines derived from BCG [40]. In fact, we observed significant skin test reactivity one month post-vaccination of cattle with either BCG or Δ*leuD* BCG-85B. Importantly, this positive reactivity in the animals vaccinated with Δ*leuD* BCG-85B decreased three months post challenge to values significantly lower than that of the nonvaccinated group, although no correlation was found between severity disease and skin test reactivity in all challenged animals.

The development of diagnostic tests that allow differentiating vaccinated from infected animals (DIVA test) is a prerequisite to use BCG-based vaccination against tuberculosis in cattle. Recently, Vordermeier et al. [41] defined combinations of *M. tuberculosis* complex antigens for DIVA diagnosis, which included ESAT6 and CFP10 antigens. Later on, Flores-Villalva et al. [42] assessed the effectiveness of an ESAT6-CPP10 antigen cocktail as an intradermal diagnostic test in field assays and found that this antigen combination allows identification of an important proportion of animals that PPDB was not able to recognize, especially in low-prevalence herds. The fact that ESAT6 and CFP10 are absent from BCG, and in turn from the Δ*leuD* BCG-85B candidate vaccine, allows the use of these both immunodominant antigens as DIVA reagents (either in an intradermal test or in the Bovigam IFN-γ assay) in combination with the Δ*leuD* BCG-85B vaccine, as part of a program to control bTB.

Given that the immune responses between bovines and humans have been considered a determinant issue in the development of diagnostic tools and vaccines both for human and bovine tuberculosis [43], the results presented here make this recombinant BCG an attractive candidate vaccine to be tested against both bovine and human tuberculosis.

## Supporting Information

Material S1 **Protective efficacy as measured by gross pathology in lymph nodes.** Mean pathology scores of lymph nodes in vaccinated and nonvaccinated groups. Pathology scores for individual animals are plotted. Horizontal lines indicate median values.
(DOCX)

Material S2 **Protective efficacy as measured by gross pathology (A) and histopathology (B and C) in lungs.** (A) Arrows indicate lesions. Only one animal from Δ*leuD*BCG-85B-vaccinated group presented an small lesion in lungs. (B and C) Images of haematoxylin and eosin stained lung sample; images 4X. Nonvaccinated and BCG groups showed advanced stage granuloma. Arrow indicate type I granuloma from Δ*leuD*BCG-85B-vaccinated group. (C) pneumonia.
(DOCX)
