# Supplementary material for: Vaccination with a BCG Strain Overexpressing Ag85B Protects Cattle against Mycobacterium bovis Challenge
Source: PLoS One. 2012 Dec 10;7(12):e51396. doi: 10.1371/journal.pone.0051396 (PMC3519572; doi:10.1371/journal.pone.0051396)
Supplement: Material S1 — Protective efficacy as measured by gross pathology in lymph nodes. Mean pathology scores of lymph nodes in vaccinated and nonvaccinated groups. Pathology scores for individual animals are plotted. Horizontal lines indicate median values. (DOCX) [file pone.0051396.s001.docx]

**Supplementary material S1**


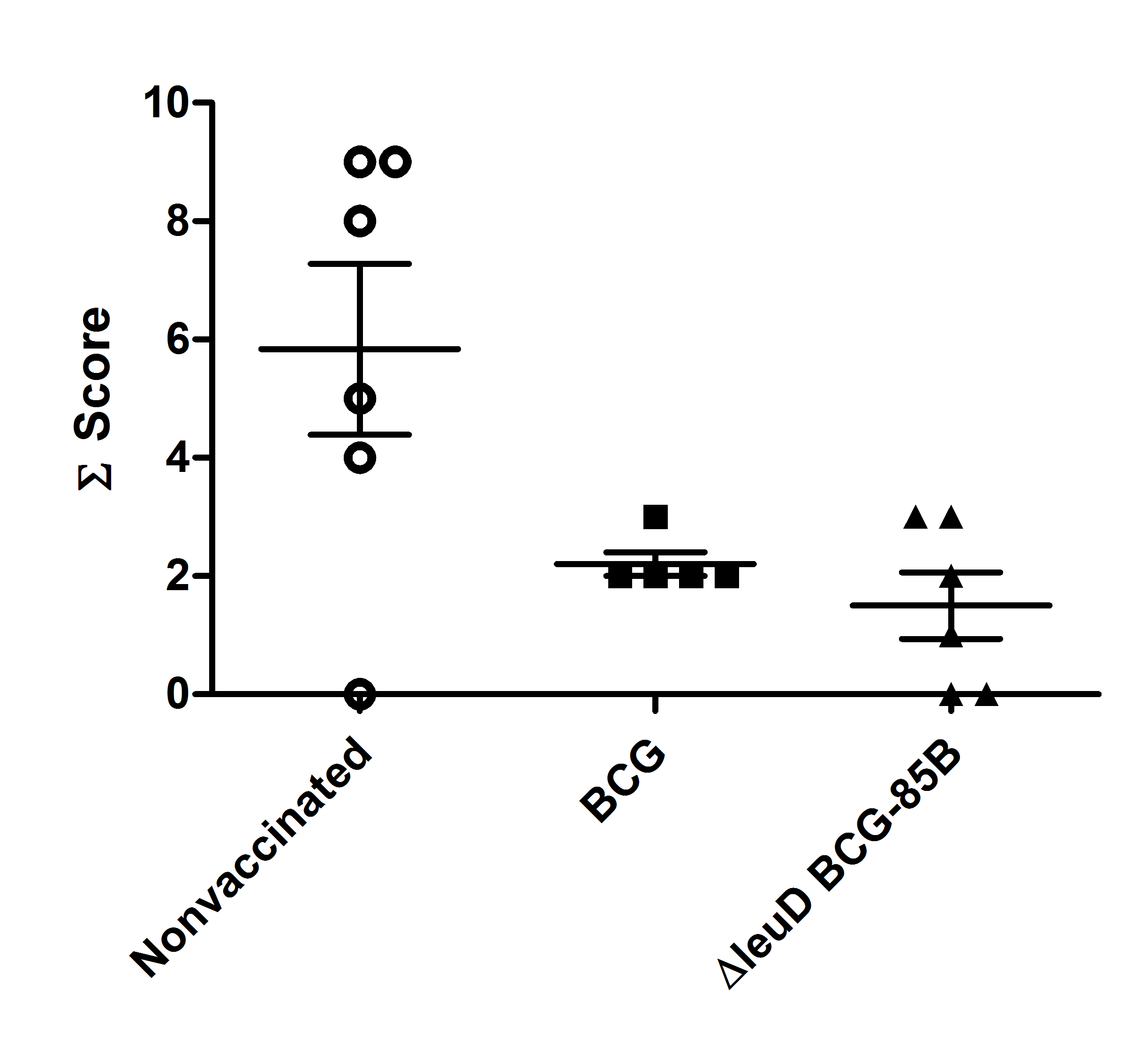


**Supplementary material S1**. **Protective efficacy as measured by gross pathology in lymph nodes**. Mean pathology scores of lymph nodes in vaccinated and nonvaccinated groups. Pathology scores for individual animals are plotted. Horizontal lines indicate median values.
