# Supplementary material for: Vaccination with a BCG Strain Overexpressing Ag85B Protects Cattle against Mycobacterium bovis Challenge
Source: PLoS One. 2012 Dec 10;7(12):e51396. doi: 10.1371/journal.pone.0051396 (PMC3519572; doi:10.1371/journal.pone.0051396)
Supplement: Material S2 — Protective efficacy as measured by gross pathology (A) and histopathology (B and C) in lungs. (A) Arrows indicate lesions. Only one animal from ΔleuDBCG-85B-vaccinated group presented an small lesion in lungs. (B and C) Images of haematoxylin and eosin stained lung sample; images 4X. Nonvaccinated and BCG groups showed advanced stage granuloma. Arrow indicate type I granuloma from ΔleuDBCG-85B-vaccinated group. (C) pneumonia. (DOCX) [file pone.0051396.s002.docx]

**Supplementary material S2**


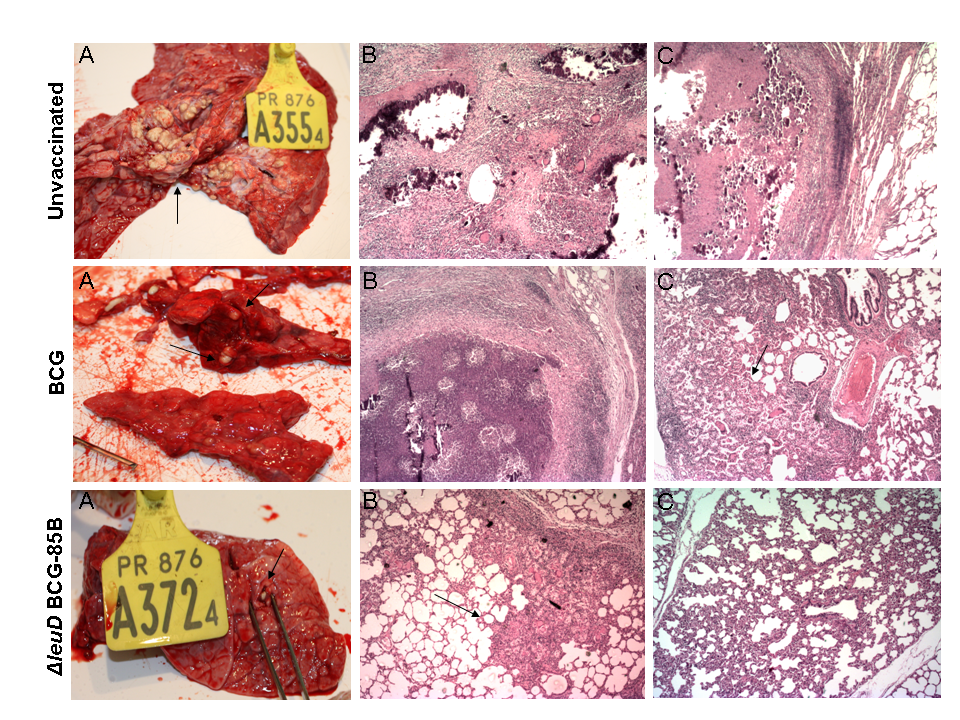


**Supplementary material S2. Protective efficacy as measured by gross pathology (A) and histopathology (B and C) in lungs**. (A) Arrows indicate lesions. Only one animal from Δ*leuD*BCG-85B-vaccinated group presented an small lesion in lungs. (B and C) Images of haematoxylin and eosin stained lung sample; images 4X. Nonvaccinated and BCG groups showed advanced stage granuloma. Arrow indicate type I granuloma from Δ*leuD*BCG-85B-vaccinated group. (C) pneumonia.
